# The Effect of Alkali Treatment on Physical, Mechanical and Thermal Properties of Kenaf Fiber and Polymer Epoxy Composites

**DOI:** 10.3390/polym13122005

**Published:** 2021-06-19

**Authors:** Nur Farhani Ismail, Nabilah Afiqah Mohd Radzuan, Abu Bakar Sulong, Norhamidi Muhamad, Che Hassan Che Haron

**Affiliations:** Department of Mechanical and Manufacturing Engineering, Faculty of Engineering & Built Environment, Universiti Kebangsaan Malaysia, Bangi 43600, Malaysia; nurfarhaniismail@yahoo.com (N.F.I.); norhamidi@ukm.edu.my (N.M.); chase@ukm.edu.my (C.H.C.H.)

**Keywords:** kenaf composites, compression moulding, physical properties, mechanical properties

## Abstract

The use of kenaf fiber as a reinforcement material for polymer composites is gaining popularity, especially in the production of automotive components. The main objective of this current work is to relate the effect of alkali treatment on the single fiber itself and the composite material simultaneously. The effect of temperature condition during mechanical testing is also investigated. Composite materials with discontinuous natural kenaf fibers and epoxy resin were fabricated using a compression moulding process. The epoxy composites were reinforced with 50 wt% untreated and treated kenaf fibers. The kenaf fiber was treated with NaOH solution (6% by weight) for 24 h at room temperature. Kenaf fiber treated with NaOH treatment had a clean surface and no impurities. For the first time we can see that alkali treatment had a damaging effect on the mechanical properties of kenaf fibers itself and the treated kenaf/epoxy composites. The composite reinforced with untreated kenaf fiber and treated kenaf fiber showed increased tensile strength (72.85% and 12.97%, respectively) compared to the neat epoxy. Reinforcement of the composite with treated kenaf fiber decreased the tensile strength due to the fiber pull out and the formation of voids which weakens the adhesion between the fibers and matrix. The temperature conditions also play an important role in composites with a significant impact on the deterioration of composite materials. Treated kenaf fiber has thermal stability and is not sensitive to temperature and as a result reinforcement with treated kenaf gives a lower loss value of 76%.

## 1. Introduction

Nowadays, plastic composites reinforced with natural fibers are well established regarding to produce unique composites with useful properties [1]. Natural fibers are widely accepted in different industries and also in the research community as reinforcement materials due to their flexibility and strength properties. Composite materials based on natural fibers are increasingly being used to replace conventional composite materials based on synthetic materials such as carbon and glass fibers [2]. This replacement is due to the fact synthetic fibers are generally expensive, harmful and not environmentally friendly [3]. While, composite materials based on natural fibers have advantages such as low cost, high specific strength and abundant resources [4].

**Figure 1:**
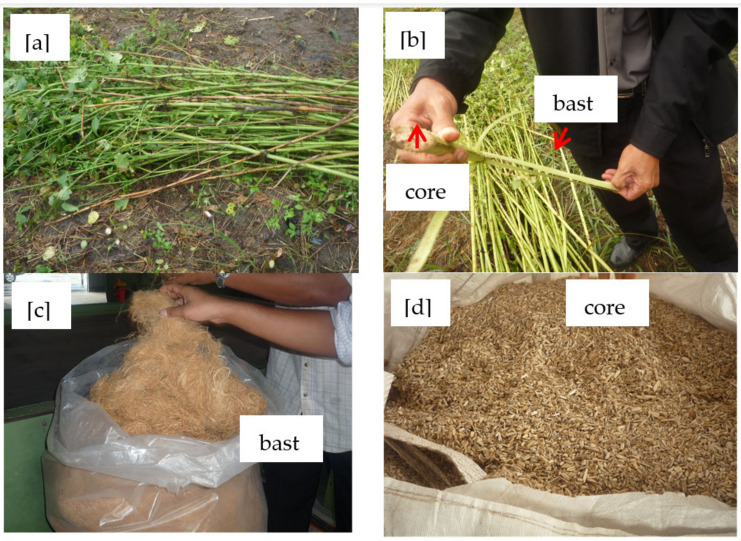
(**a**–**d**). Picture of kenaf fiber.

Compared to synthetic fibers natural fibers mostly have lower strength and durability, but natural fibers have low specific gravity which results in a high specific strength and stiffness compared to synthetic fibers [5]. There are variety of natural fibers such kenaf, sisal, hemp, flax, abaca, pineapple leaf, and ramie that have been widely used during the human evolution [6]. Among these natural fibers, kenaf (*Hibiscus cannabinus*) fiber was chosen as a reinforcement material in this study because it is planted commercially. The cheap, lightweight and biodegradable kenaf fiber material also has good mechanical properties compared to other natural fibers [3] Kenaf plants (Figure 1a,b) are also expansively planted in Malaysia by the Tobacco Board of Malaysia (LKTN). Furthermore, this plant can grow under a wide range of weather conditions. Generally, kenaf plants consist two type of fibers; (1) 60–65% core fiber and (2) 35–40% bast fiber which is outer fibrous bark surrounding the core (Figure 1b–d) [7].

Nowadays, numerous studies conducted around the world have proved that kenaf can be used to produce kenaf-based composites with acceptable physical and mechanical properties. Kenaf fiber composites are being increasingly used in many applications in the aircraft, aerospace, automotive, marine and other industries [8]. This is due to their numerous desirable physical and mechanical properties including high specific strength and high specific stiffness [9,10]. Furthermore, kenaf fiber composites are attractive structural materials due to their advantages such as environmental friendliness, acceptable biodegradability, low density and price [11,12]. However, previous works have reported that kenaf fiber composites are vulnerable to heat and moisture when used under changing environmental conditions which has a tendency to influence the properties of the fiber itself [13,14]. Furthermore, kenaf fiber composites also have other limitations including incompatibility between the hydrophilic natural fibers and hydrophobic polymers.

Among the numerous factors that influence the performance of the polymer composites reinforced with natural fibers is the resulting chemical modification [15]. This modification can improve the adhesion between the matrix and fiber and this tends to influence the mechanical and thermal properties of the fiber-reinforced composites [16]. Several chemical treatments such as bleaching, acetylation and alkali treatment for improving fiber matrix interfacial bonding have been suggested by many researchers [17]. These chemical treatments can be good candidate to overcome this issue by improving the natural fiber–matrix interfacial adhesion [18]. It has been proposed that modifying the fiber surface and modification of the polymer matrix can improve the compatibility of hydrophobic polymer and hydrophilic cellulose fiber.

In order to reduce the incompatibility of the two phases in the composite, the most popular treatment, which is alkali treatment, was applied to natural fibers aiming to reduce their hydrophilic nature and also improve the adhesion between the fiber and the matrix [19]. Alkali treatment, represented in Equation (1), is also known as mercerization and can remove the lignin, hemicellulose, wax and oils that cover the fiber surface and also reduce the diameter of fibers as well [14,20].

Fiber − OH − NaOH → Fiber − O − Na + H_2_O (1)

Furthermore, this alkali treatment leads to the development of surface roughness which results in an increased in the amount of cellulose exposed on the surface of fibers, resulting in better interfacial adhesion between kenaf and the polymer and increasing the number of bonds between the polymer and the fiber surface [21].

Previous work has reported the use of various concentrations of alkali treatment such as 3 wt%, 6 wt% and 9 wt% [22]. It was revealed that 6 wt% of NaOH was the optimum and most effective concentration for cleaning the fiber surface, resulting in good tensile properties [23]. Previous research by Mutasher et al., revealed that alkali treatment enhanced bonding between the matrix and fiber for brittle-fractured composites. Besides that, increases of up to 10% for tensile modulus, 5% for tensile strength and 6% for flexural strength were observed for an epoxy composite reinforced with treated kenaf fiber. This result is supported by the work of the Fiore et al., group who found that reinforcement with treated kenaf increased the mechanical properties of composites due to good adhesion of the treated kenaf [19].

Basically, the polymer matrix is an appropriate medium which functions to hold the fiber together, transfer the load to the fiber reinforced composites and besides acts as a coating and adhesive material to protect the composites from any environmental factors and mechanical damage [24,25]. The most common conventional polymer matrices can be divided into two groups; thermoplastics (polypropylene, thermoplastic polyurethane) and thermosets (polyurethane, polyester, and epoxy).

Among these polymers, epoxy resin was selected and used in the fabrication of composites based on natural fibers in order to produce large composite material parts with acceptable physical and mechanical properties. Among the good features of this attractive epoxy matrix are good thermal and chemical resistance, good dimensional stability and high tensile properties (strength and modulus) [24]. Selection of a suitable fabrication process for a composite is crucial regarding the production of desired products with good quality. Epoxy composite reinforced with natural fiber can be manufactured easily using a compression moulding process compared with other manufacturing processes. Compression moulding has attractive features which are low cycle time and high reproducibility [6].

The properties of the kenaf fiber composites not only depend on the fiber itself, but also on the temperature exposure during mechanical processing [26], so researchers have investigated the thermal degradation of polymer matrix composites as this is a major problem in the application of thermoset polymers in different types of environments. Composite material behavior at different temperatures is an important parameter to be considered to determine which temperature produces the most appropriate material properties.

The type of fiber is one of the main conditions that affect the final composite product. In this work, a randomly oriented kenaf mat was chosen as reinforcement material due to its properties which can be equal to those of the synthetic fiber Kevlar [6]. As compared to an unidirectional kenaf mat, a randomly oriented fiber mat can provide superior resistance to impact. In this present study we also focus on the effect of alkali treatment on the kenaf fiber itself and kenaf/epoxy composites. As mentioned by previously by other researchers, 6 wt% NaOH concentration was considered as an effective chemical treatment which can remove all the impurities on the surface of natural fibers [23]. However, it is still less exploited because the final result depends on the type of kenaf bast used. Nevertheless, in this work 6 wt% NaOH was selected as chemical treatment for this type of kenaf fiber in a novel application. This study also sought to evaluate the effect of temperature conditions on the tensile properties of kenaf/epoxy composites when the tensile tests were performed at different temperatures.

## 2. Materials and Methods

### 2.1. Materials

The discontinuous long temafa kenaf fiber with density 1.71 g/cm^3^ and diameter in between 45 to 250 μm was obtained locally from Lembaga Kenaf Tembakau Negara (LKTN, Kubang Kerian, Kota Bharu, Malaysia). An epoxy resin matrix (D.E.R^TM^ 331^TM^ ) was used as the base polymer matrix and combined with the curing agent and the hardener JOINTMINE^TM^ 925-3S^TM^. The compression grade epoxy and hardener were provided by Dow Chemical Company (Midland, MI, USA) and Epochemie International Pte Ltd. (Singapore, Singapore), respectively. Table 1 summarizes the mechanical properties of the epoxy and hardener according to the respective MSDS.

### 2.2. Fiber Modifications

Alkali treatment of kenaf fiber was carried out to remove surface hemicelullose and impurities [19]. Sodium hydroxide (NaOH, Merck, Darmstadt, Germany) with 6 wt% concentration was used to promote better interaction between the kenaf fiber and polymer matrix. Kenaf fibers were soaked in NaOH solution for 24 h at room temperature. The fibers were then immersed in tap water containing 1 wt% of glacial acetic acid (EMSURE, Wiesbaden, Germany) to neutralize the excess of NaOH, followed by a soaking process for 30 min in distilled water [27]. Then, treated kenaf fibers were washed with distilled water until the pH reached a value of 7 and then dried in an oven environment at 40 °C for 24 h prior to the composite fabrication [23]. Thermogravimetric analysis (TGA), dynamic differential scanning calorimetry (DSC), Fourier transform infrared (FTIR) spectroscopy, tensile strength and scanning electron microscopy (SEM) were used to characterize the properties of treated and untreated kenaf fibers.

### 2.3. Characterizations of Untreated and Treated Kenaf Fibers

TGA analysis was conducted using a STA 449F3 Jupiter system (Netzsch, Barcelona, Spain) to observe changes in temperature and time in a controlled environment. TGA was often used to figure out how much the weight of the mixture changed due to oxidation or decomposition. TGA patterns were observed in a nitrogen atmosphere from room temperature to 700 °C at a heating rate of 10 °C/min.

Differential scanning calorimetry was used to examine the thermal properties of the untreated and treated kenaf fibers. Samples (12.83 mg) of untreated and 15.45 mg of treated kenaf fiber were placed on an alumina crucible pan under a nitrogen atmosphere. The analysis was conducted started from room temperature up to maximum temperature at 700 °C with heating rate 10 °C/min under nitrogen environment.

FTIR analysis was performed with using a 6700 ATR-FTIR spectrometer (Thermo Scientific Nicolet, Waltham, MA, USA). All the spectra were recorded with wavelength between 450 cm^−1^ until 4000 cm^−1^. FTIR patterns were used to determine molecular orientation, fiber structure and the changes before and after fiber degradation for the untreated and treated kenaf fibers.

**Figure 2:**
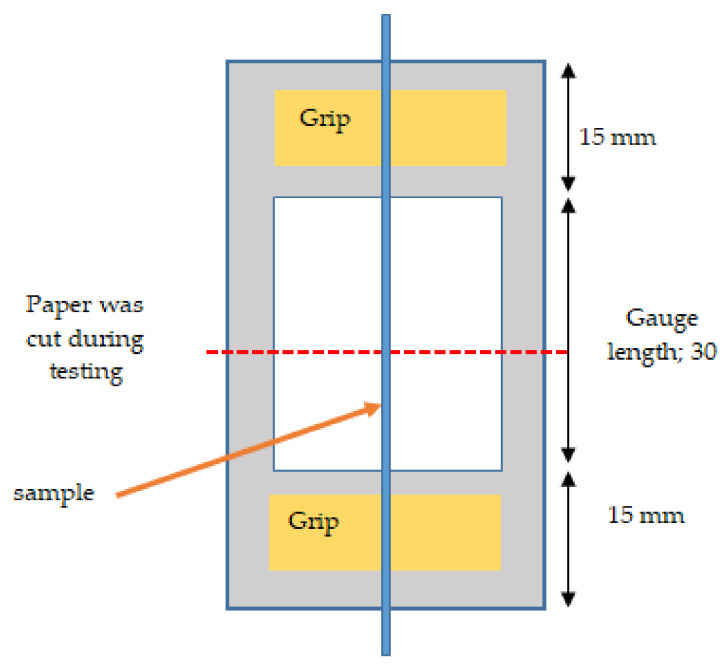
Schematic layout of the single kenaf fiber.

The tensile strength and Young’s modulus of the five replicate single fibers was measured using a model 3365 universal testing machine (Instron, Norwood, MA, USA) with a specific grip method with 5 kN load cell as shown in Figure 2. This testing was conducted according to the ASTM D3379 instructions. Due to the sensitivity of the single fiber, a grip made of thin paper was designed with a hole that can be attached to the upper hook of the universal testing machine. The single fiber was bonded with tape to the paper in the grip for the the better gripping and then 30 mm fiber was stretched at a rate cross speed of 0.1 mm/min. The fiber diameter was obtained using an optical microscope with camera (image analyzer) and diameter value was calculated as an average of the best three readings. The standard specifies that all the tests be carried out on single fibers at the effective gauge length of 30 mm and under room temperature (±27 °C) conditions.

A VP-SEM SU1510 pressure scanning electron microscope (SEM, Hitachi, Hitachi High Technologies America, Inc., Irving, TX, USA) was used to examine the effect of the NaOH treatment by observing the surface morphology of kenaf bast fiber. Prior to that, the samples were coated with gold then finally viewed under SEM to examine and identify impurities on the surface of the treated kenaf fibers.

### 2.4. Fabrication of Epoxy Composites Reinforced with Kenaf Fibers Using Compression Moulding

**Figure 3:**
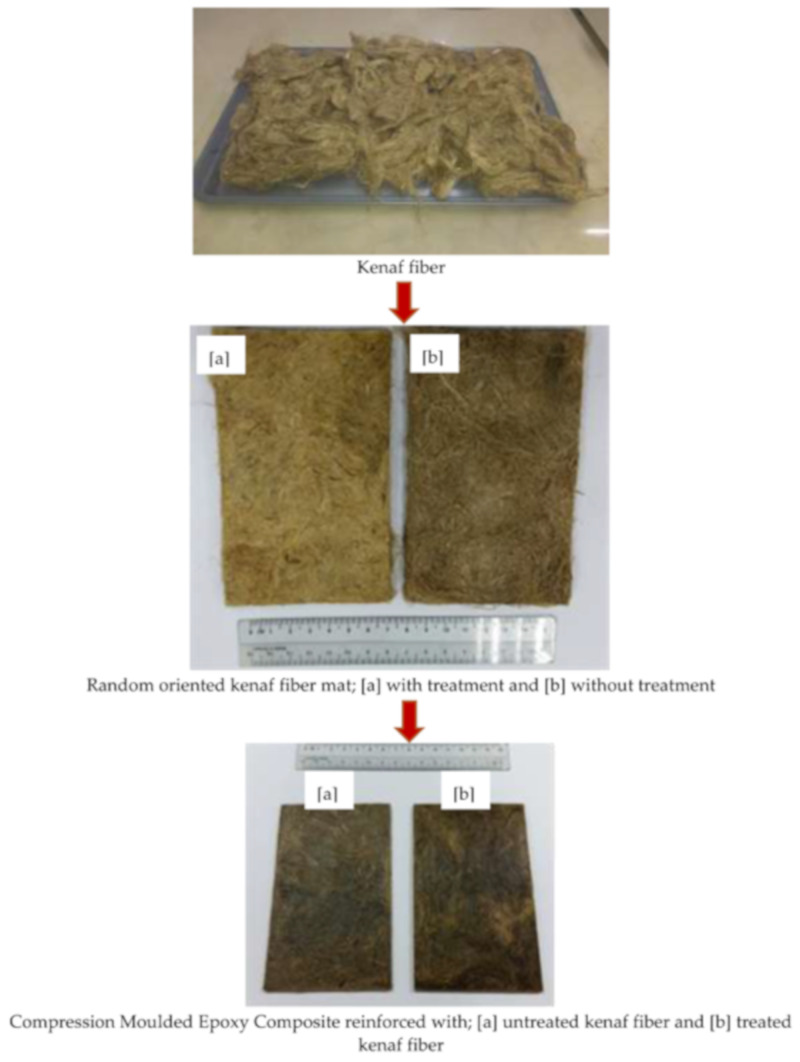
Compression moulded epoxy composites reinforced with untreated kenaf fiber and treated kenaf fiber.

The samples were prepared using a compression moulding process as shown in Figure 3. The sample preparation was carried out in two stages: (1) pressing the kenaf fiber without polymer matrix and (2) fabrication of kenaf/epoxy composites. In the first stage prior to the composite fabrication process, 50 wt% of kenaf fibers without matrix were pressed at 10 MPa for 5 min using a compression moulding machine to make a randomly oriented fiber mat [28]. The epoxy and hardener were then combined in a 2:1 ratio with a mechanical stirrer at 2 rpm for 5 min before being cast into the mould with the kenaf mat. These parameters were used to prevent bubbles and also to prevent the resin from becoming a gel. For the pre-pressing process, the mould was placed in the compression moulding environment for 15 min. The mould was then pressed at 10 MPa of compression moulding for 25 min. The specimen was then post-cured for 20 min at room temperature after being removed from the compression mould for the cooling process and the fabricated composite was kept in a dry cabinet in order to avoid moisture before cutting a process sample following standard testing procedures [29]. The mechanical properties such as tensile strength and dynamic mechanical behaviour were tested on the epoxy composite reinforced with untreated and treated kenaf fibers. Also, SEM of the fracture surface of the untreated and treated kenaf/epoxy composites were carried out. Before all the characterizations and measurements were carried out, the samples need to be dried in an oven to reduce the moisture.

### 2.5. Characterization of Epoxy Composite Reinforced with Untreated and Treated Kenaf Fibers

Tensile tests were carried out using an Instron 5567 Universal Testing Machine Model with a 30 kN load cell attached to a chamber with the set temperature. The tensile strength and tensile modulus of the untreated and treated kenaf/epoxy composites were determined according to the ASTM D3039 standard. A crosshead speed of 2 mm/min and a gauge length of 65 mm was used for carrying out the test. Five samples of composites with dimensions 115 mm (length) × 20 mm (width) × 2 mm (thickness) were tested under different temperatures (room temperature, 50 °C, 75 °C, 100 °C, 125 °C and 150 °C). The tensile tests were conducted until tensile failure occured. Five replicate samples of each group were taken and the average reading data value was reported. Also, the standard deviations values were included for each test.

Dynamic mechanical analysis (DMA) was conducted to determine the glass transitions and mechanical properties of neat epoxy and epoxy composites reinforced with untreated and treated kenaf fibers under the process control temperature. Samples were cut into 30 mm (length) × 10 mm (width) × 2 mm (thickness) before were subjected to dynamic mechanical tests. A model DMA8000 dynamic mechanical analyzer (Perkin Elmer Corp, Houston, TX, USA) was used to determine the storage modulus (E′), loss modulus (E″) and damping factor (tan δ) of the composites. Single cantilever bending modes were applied in this study. The samples were measured over a wide range temperature, from room temperature (±27 °C) up to 200 °C at a heating rate 2 °C/min. The samples were analyzed at a fixed frequency (1 Hz) and static strain (0.02 or 0.2%).

The interfacial surface of the epoxy composite reinforced with untreated and treated kenaf fiber was also studied. The fractured tensile surfaces of the composite samples were dried prior to coating with platinum sputtered for 120 s. Then, the microstructures of samples were analyzed using a FESEM SUPRA 55VP system (Carl Zeiss, Oberkochen, Germany). 

## 3. Results and Discussion

### 3.1. Thermal Stability Analysis

**Figure 4:**
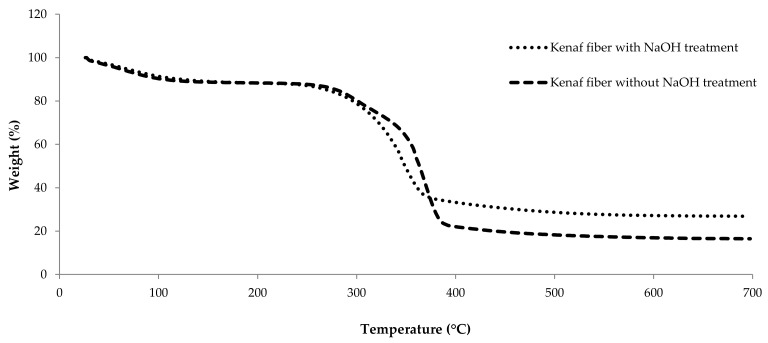
Thermogravimetric analysis (TGA) curves for kenaf fiber with NaOH treatment and without NaOH treatment.

In general, a major limitation to using natural fibers as reinforcements in polymer matrix composites is their low thermal stability. The thermal stability and thermal degradation of untreated and treated kenaf fibers were determined from the corresponding TGA traces. The TGA results basically display a curve of the weight loss variation with temperature. Figure 4 shows the TGA thermograms of both types of kenaf fibers in a nitrogen atmosphere. Studies have reported that natural fibers are sensitive to temperature and complete thermal degradation is expected to occurred at temperatures of 400 °C and above [16]. The untreated kenaf fiber begins to lose weight earlier than kenaf fiber with treatment. This may be due to the higher moisture content of untreated fiber, as well as the presence of hemicellulose, which allows the composite to retain more moisture [23,30].

All the thermogravimetric analysis plot results are summarized in Table 2. The degradation of the kenaf fibers exhibits three stages: Based on the graft, a slight mass loss was observed below 200 °C which may be attributed to evaporation of absorbed moisture. Theoretically, the moisture starts to evaporate at a temperature of 80 °C because it is difficult to totally eliminate the water even though the kenaf was dried in an oven before running the TGA analysis [16,31]. Table 2 shows that treated fibers have less mass loss than untreated kenaf fibers. The results indicate that the evaporation of moisture and maximum degradation of the treated and untreated kenaf fibers occurs at 89.93 °C and 79.89 °C. The graph also illustrates that hemicellulose was decomposed within a temperature range of 200–400 °C. This finding supports the results of the Azwa et al., group where they revealed that hemicellulose decomposed within temperature range of 135–400 °C [23]. Compared to the untreated kenaf fiber, the degradation peak of the treated kenaf fibers occurred at higher temperatures, indicating that treated kenaf fiber exhibited higher thermal stability at higher temperatures.

**Figure 5:**
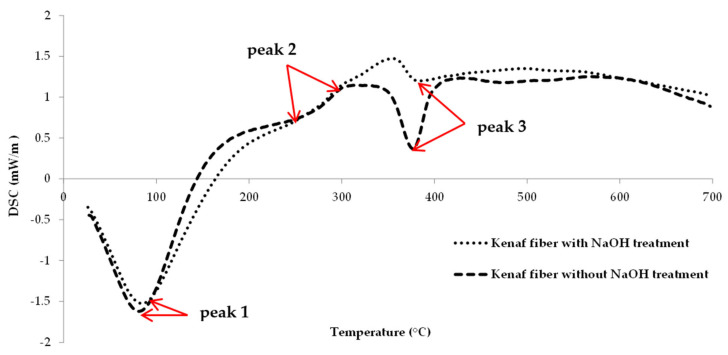
DSC curves for kenaf fiber with NaOH treatment and without NaOH treatment.

The DSC curve for the epoxy composite reinforced with untreated and treated kenaf is shown in Figure 5. It is clearly shown that there are three peaks for both types of kenaf fiber. Each peak represents the temperature of the maximum rate of weight loss occurred which is the critical temperature at which the kenaf fiber decomposes. By referring to the DSC thermograms, each of the peaks refers to the compound of the fiber; peak 1 refers to moisture content, peak 2 refers to hemicellulose and peak 3 refers to cellulose content and each is also summarized in Table 3. The extended temperature range that starts at 400 °C up to 700 °C represents the lignin content. From that, it can be concluded that the untreated kenaf fiber decomposed earlier compare to treated fiber. Previous studies have mentioned and proved that alkali treatment increases the thermal stability of the fibers [32].

### 3.2. Tensile Properties of Kenaf Single Fibers

The results showing the effects of NaOH treatment on the tensile strength and Young’s modulus of the natural fiber can be viewed in Table 4. Tensile strength and Young’s modulus of the fiber itself are considered important properties which can estimate the tensile properties of the composite. The results show that the tensile strength and Young’s modulus of the kenaf fiber seems to drop after 24 h of treatment. By considering and observing the results in Table 4, some speculations from the previous literature on the effect of NaOH treatment on the tensile strength can be made, which is breakage of the fiber during the alkalization step [33]. Alkali treatment resulted in the removal of lignin, hemicellulose and wax which contributes to the deterioration of the mechanical strength properties of kenaf fibers such as tensile strength [34].

### 3.3. FTIR Spectroscopy Result

**Figure 6:**
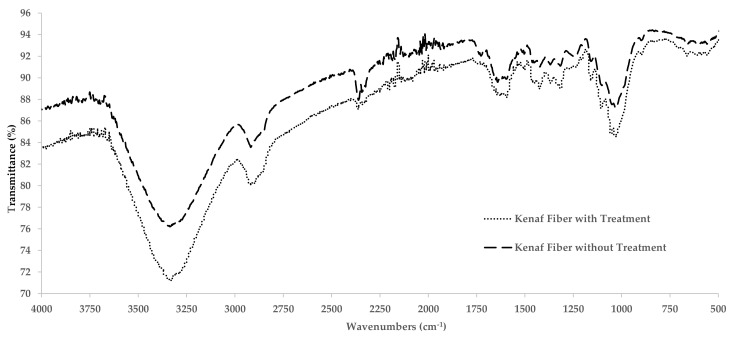
FTIR spectroscopy results of untreated and treated kenaf fibers.

FTIR spectra display the intensity of infrared bands and wavenumbers which represent the molecular bonding. Figure 6 illustrates the FTIR spectra for kenaf fibers with NaOH and without NaOH treatment. The aim of the experiment is to see the effect of NaOH treatment on the kenaf fiber as well as to determine the cellulose and hydroxyl groups. Overall, the results show that untreated and treated kenaf fiber have differences in terms of the band intensity. Based on the graph a wide band appeared around 3300 cm^−1^ which confirms the presence cellulose molecules with free form OH groups while the band appearing around 2900 cm^−1^ corresponds to the C-H absorption band. The results for the treated kenaf fiber show the disappearance of the peak around 1700 cm^−1^ which confirms the elimination of lignin and hemicellulose after the NaOH treatment [35,36]. Meanwhile the peak identified for untreated kenaf fiber at 1730 cm^−1^ was attributed to the presence of C=O which represents the groups in hemicellulose and lignin [28].

### 3.4. Morphological Analysis for Kenaf Fibers

**Figure 7:**
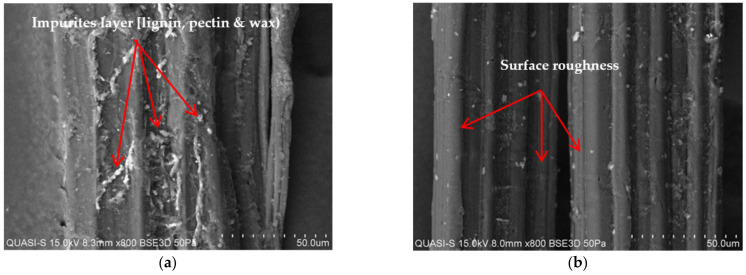
SEM images from longitudinal views for kenaf fibers; (**a**) untreated kenaf fiber; (**b**) treated kenaf fiber.

The surface morphologies of untreated and treated kenaf fiber are displayed in Figure 7a,b. Comparison between untreated and treated kenaf proves that alkali treatment has an effect on the surface of the kenaf fiber. Structure images of untreated kenaf fiber are not smooth and have irregular stripes due to the existence of impurities such as lignin, pectin, wax, etc that cover the surface of the fiber as shown in Figure 7a, while in Figure 7b, the physical appearance shows that treated kenaf fiber had a clean, clear and rough surface after alkaline treatment. This treatment is an essential stage in natural fibers in order to remove the hemicellulose, lignin and wax from the outer surface of the kenaf fibers, which enhances the interfacial bonding between the kenaf and the polymer matrix. This is supported by the research results of Masitah et al. who stated that the surface of alkali-treated fiber has no impurities and this alkali solution increased the mechanical bonding between the fiber and matrix polymer [37].

### 3.5. Tensile Strength Properties of Untreated and Treated Kenaf/Epoxy Composites

**Figure 8:**
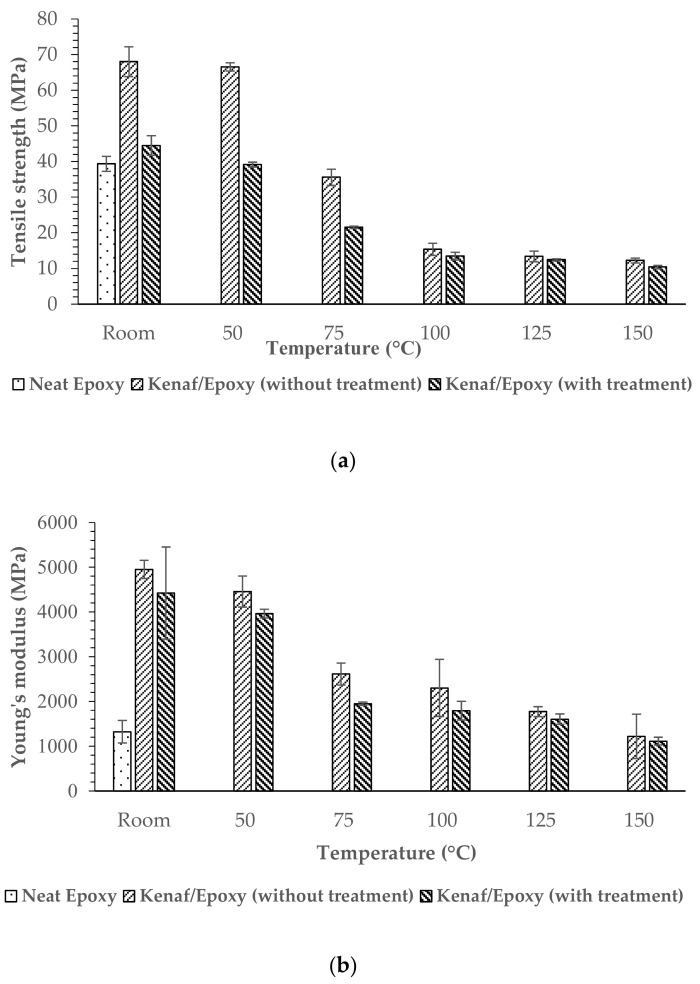
(**a**) Tensile strength and (**b**) Young’s modulus of untreated and treated kenaf/epoxy composites.

**Figure 9:**
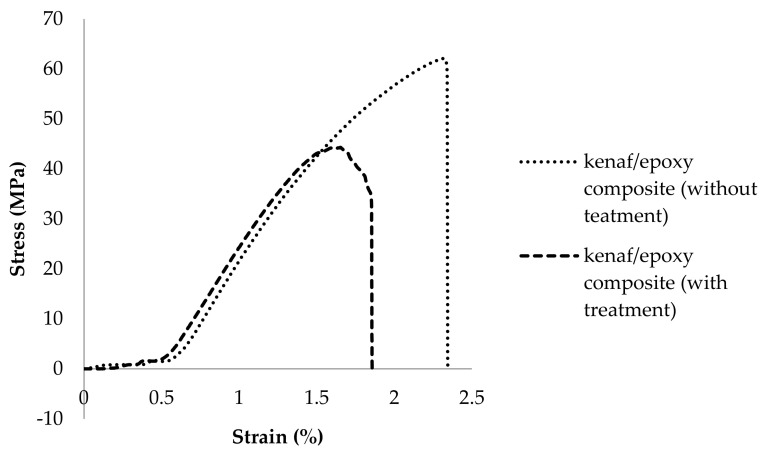
Stress-strain curves of untreated and treated kenaf/epoxy composites obtained from tensile test curves.

The tensile strength and the Young’s modulus of epoxy composites reinforced with untreated and treated kenaf fiber composites are presented in Figure 8a,b. The results revealed in the graph are similar to those of the tensile fiber itself described in Section 3.2. In this study it was found that epoxy composite reinforced with untreated kenaf fiber has higher tensile strength (68 MPa) at room temperature. A similar trend also can be seen in Figure 8b where the Young’s modulus of epoxy composite seems to increase when reinforced with untreated kenaf fiber. It is worth noting that the treatment of kenaf fibers in NaOH solution influences the tensile properties of composites. Figure 9 show the stress-strain curve of the tensile stress. Based on the graph, a linear curve typically shows that both materials have brittle behavior. The epoxy composite reinforced with treated kenaf fiber generally broke before the epoxy composite reinforced with untreated kenaf fiber. This shows that reinforcement of untreated kenaf fiber in composite materials tends to produce brittle materials, while, the reinforcement of untreated kenaf fiber in composite produced a high tensile stress with a high strain value (2.34%).

The results show that reinforcement with treated kenaf fiber slightly decreases the mechanical properties of the resulting epoxy composite. Nevertheless, as discussed above in Section 3.2, the alkaline treatments decrease the mechanical properties of the fiber itself. However, reinforcement of untreated kenaf increased tensile strength 72.8% more than neat epoxy at the average of 39.3 MPa. Incorporation of the treated kenaf fiber increased the tensile strength by 12.97%. The same trend also was recorded for the tensile-modulus strength where the incorporation of the kenaf fiber either untreated or treated increased the mechanical strength compared to neat epoxy. The decrease in tensile strength of the epoxy composite reinforced with treated kenaf fiber is because the NaOH solution damages the fiber texture which leads it to be more fine and brittle. A detailed explanation and support will be discussed in Section 3.7.

Figure 8a,b present the strength decrease of untreated and treated kenaf/epoxy composites over the entire range of test temperatures. Epoxy polymer composites presented a strong and marked temperature dependency. In the analysis, the epoxy composite consistency decreased upon increasing the test temperature. The loss computed in the treated kenaf/epoxy is smaller than that of the untreated kenaf/epoxy composite. The tensile strength loss of the treated kenaf/epoxy was 76% when the temperature reaches 150 °C as compared to room temperature samples. Meanwhile, the flexural strength of the untreated kenaf/epoxy gradually decreased with a 82% loss between room temperature and 150 °C.

### 3.6. Dynamic Mechanical Properties of Untreated and Treated Kenaf/Epoxy Composites

**Figure 10:**
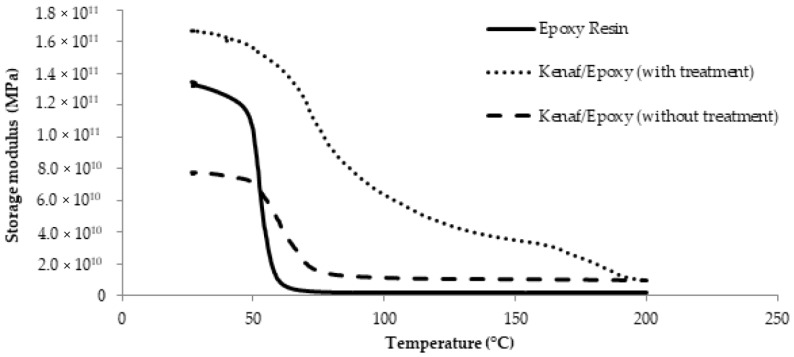
Storage modulus, *E*′ spectra for epoxy composite reinforced with untreated and treated kenaf fiber.

Behaviors and characteristics of polymer composite materials can be studied by dynamic mechanical analysis (DMA) which is a powerful, quick and easy tool for material properties measurement [38]. Basically, the storage modulus (*E*′) narrowly interrelated to the bearing capacity of materials and also related to the flexural modulus which is measured following ASTM D790 [15]. Storage modulus spectra for untreated and treated kenaf/epoxy composite are presented in Figure 10 where it is found that the storage modulus decreased with increasing temperature. In general, spectra of storage modulus versus temperature deliver information about stiffness properties, fiber/matrix interfacial bonding and crosslinking of the materials. Clearly it can be seen that at low temperature (room temperature), the storage modulus values were minimum when the the epoxy composites were reinforced with untreated kenaf fiber. However, the result is different when treated kenaf fiber was added and one can observe an improvement in the storage modulus value. Figure 10 also shows that the storage modulus displays a significant drop in the region between 50 °C and 75 °C. Generally, the storage modulus decreases as the temperature increases due to the loss of the close packing arrangement of the epoxy composite structure [38].

**Figure 11:**
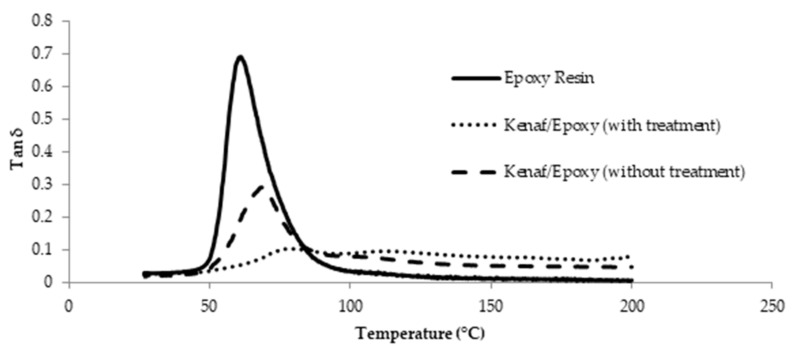
Damping factor (tan δ) spectra for epoxy composite reinforced with untreated and treated kenaf fiber.

Figure 11 shows the variations in the damping factor of the epoxy composites reinforced with untreated and treated kenaf fiber as a function of temperature. It was observed that the damping factor increased with temperature, reaching a maximum in a transition region before decreasing into a rubbery region. Neat epoxy has a higher tan δ at the glass transition temperature and remained on the top over the whole temperature range [38]. In the polymer composite materials, the damping is influenced by the fiber content and also the modification of the fiber itself [39]. The incorporation of fibers in a composite system affects damping due to the elastic nature of the fibre and shear stress concentrations at the fiber ends, in conjunction with the additional viscoelastic energy dissipation in the matrix material. This can be seen in Figure 11 in which epoxy treated kenaf/epoxy composite with lower tan δ value has different elastic properties compared to epoxy composite reinforced with untreated kenaf [40]. Theoretically, the fiber content, fiber size and fiber surface treatment affect the dynamic mechanical properties [41]. Besides, the tan δ value also depends on the fiber matrix adhesion wherein a weak fiber matrix adhesion will result in a higher tan δ value. Based on Figure 11, the reinforcement of the treated kenaf fiber in epoxy composite possessing a low tan δ value may be due to the strong bonding and good adhesion between the matrix and fiber [42].

**Figure 12:**
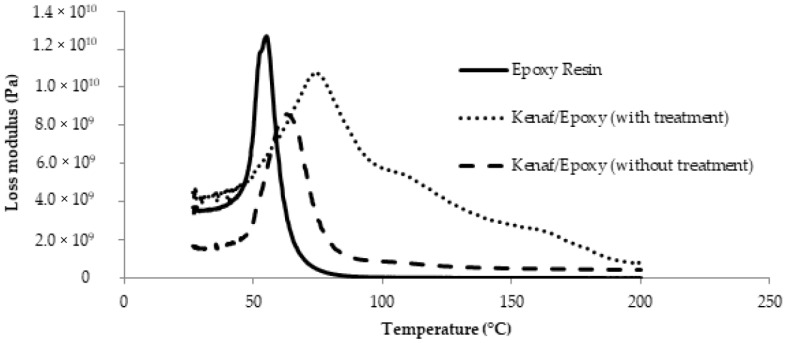
Loss modulus spectra for epoxy composite reinforced with untreated and treated kenaf fiber.

Figure 12 shows the variation trend of loss modulus spectra for untreated and treated kenaf/epoxy composite as well as neat epoxy. Figure 12 very clearly shows that reinforcement of the epoxy composite with natural fiber leads to a widening of the modulus peak compared to neat epoxy which has a narrow peak [43]. This peak widening is due to the increased number of chain segments as well as the fiber volume. This peak widening can explain the inhibition process of the composite, as it is seen that the loss modulus corresponding to the T_g_ in the epoxy composite reinforced with treated kenaf is increased compared to the neat epoxy and untreated kenaf/epoxy composite.

### 3.7. Morphological Analysis for Epoxy Composite Reinforced with Untreated and Treated Kenaf Fiber

**Figure 13:**
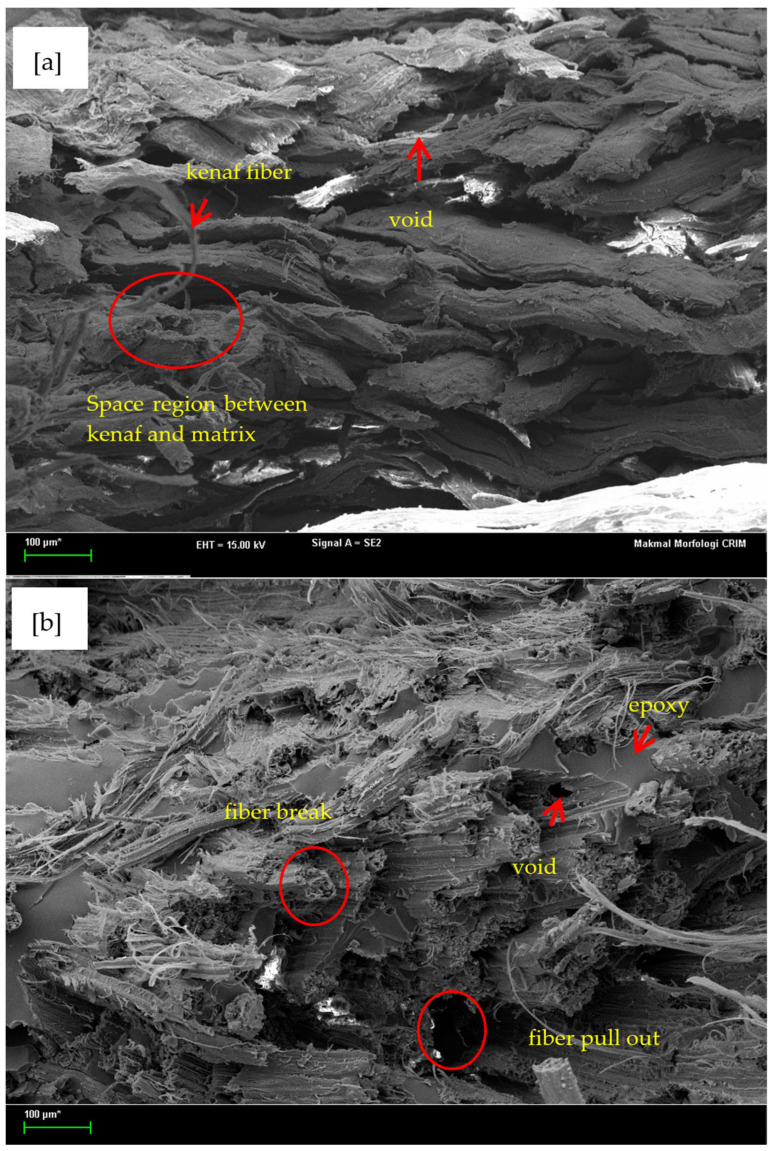
SEM image (100× magnification) of epoxy composite reinforced with; (**a**) untreated kenaf fiber and (**b**) treated kenaf fiber.

The distribution and bonding between epoxy matrix and kenaf fiber are presented and analyzed in this section. Figure 13 illustrates the SEM observations of the composites after running the tensile testing analysis in a room temperature environment. These figures give the result of the epoxy composite reinforced of the different types of kenaf fiber; with and without NaOH treatment. It was found that some voids exist in the untreated kenaf/epoxy composite as shown in Figure 13a. In addition, a close up of the bond interface between kenaf and epoxy can been in the SEM micrograph in Figure 13a where a space region (debonding region) is present between them. This is may be due to the fact the untreated kenaf fiber has a wax layer on the surface thus preventing the epoxy from enter the core of the fiber [18]. However, the situation is different for the epoxy composite reinforced with treated kenaf fiber as very clearly shown in Figure 13b. The result shows that kenaf fiber treated with alkali developed good adhesion to the epoxy composite, but as predicted, there is a failure in the composite which is the kenaf fiber breakage and pull out from the base (matrix). Unsurprisingly, this fiber breakage is obviously supported by the tensile property changes as mentioned previously in Section 3.5 where reinforcement with treated kenaf fiber decreased the tensile properties. The failure of this composite may be due to the fact the percentage of NaOH concentration or immersion time was not significant for this type of kenaf fiber. Besides, the void content influences the tensile properties of the composite. The low void contents of the composite in Figure 13b leads to a decrease of the tensile strength and Young’s modulus of the composite (see Figure 8a,b).

## 4. Conclusions

The effects of alkali treatment on kenaf fiber and epoxy composite were evaluated and can be summarized as below;

- Treated kenaf fiber exhibited higher thermal stability at higher temperatures compared to untreated kenaf fiber.
- As compared to treated kenaf fiber, untreated kenaf fiber showed high tensile properties and 6 wt% alkali treatment decreased the tensile strength by 34.65% while the Young’s modulus dropped 11.92%
- FTIR analysis show the disappearance of a peak due to the elimination of lignin and hemicellulose after the NaOH treatment.
- Immersion of kenaf fiber in 6 wt% NaOH for 24 h successfully cleans the fiber surface removing all the impurities.
- Alkali treatment has an influence regarding the increase of thermal stability of composites with increasing temperature during thermal degradation.
- Incorporated treated kenaf in the epoxy composite significantly decreases the mechanical properties of the epoxy composite.
- Epoxy composite reinforced with untreated kenaf fiber is sensitive to temperature whereby it shows a higher loss value (82%) between room temperature to 150 °C. Room temperature is the optimum temperature for the treated kenaf/epoxy composite where the tensile strength starts to become lower than a neat epoxy starting from a temperature of 50 °C onwards.
- The storage modulus was influenced by the chemical treatment and also temperature. Storage modulus values were minimum for the composite reinforced with treated kenaf fiber and decreased as the temperature increased.
- Alkali treatment leads to lower tan δ values and increases the loss modulus value.

According to the results achieved in this research, kenaf fiber is applicable in the engineering sector especially in the fabrication of polymer composites, however, further experimental research is necessary in determine the optimum concentration of NaOH depending on the type of fiber. This stage is appropriate to avoid the failure of the natural fiber itself and also the resulting composites.

## Figures and Tables

**Table 1:** Characteristics of epoxy, D.E.R^TM^ 331^TM^ and hardener, JOINTMINE^TM^ 925-3S^TM^ (MSDS sheet).

Properties	Epoxy	Hardener
Flexural strength [N/mm^2^]	96	-
Flexural modulus [kN/mm^2^]	3.0	-
Yield Compressive strength [N/mm^2^]	112	-
Tensile Strength [N/mm^2^]	79	-
Elongation at break [%]	4.4	-
Gel point time [min] 500 g	25	-
Density [g/cm^3^]	1.16	1.07 ± 0.02
Mixing ratio	2	1

**Table 2:** Thermogravimetric (TG) results for kenaf fiber.

Sample	Temperature Transition Range [°C]	Maximum Temperature Rate [°C]	Weight Loss [%]
Kenaf fiber with NaOH treatment	26–200	89.93	10.94
	200–300	258.63	20.71
	300–390	386.13	65.97
Kenaf fiber without NaOH treatment	27–200	79.89	11.67
	200–350	277.79	27.97
	350–390	377.39	79. 90

**Table 3:** DSC result for kenaf fiber.

Peak	Compound
Peak 1	moisture content
Peak 2	hemicellulose
Peak 3	cellulose

**Table 4:** Tensile strength comparison between untreated and treated kenaf fiber.

Types of Fiber	Tensile Strength [MPa]	Young’s Modulus [MPa]
Kenaf fiber with NaOH treatment	138.92	5767.53
Kenaf fiber without NaOH treatment	159.98	8201.52

## Data Availability

The data presented in this study are available on request from the corresponding author.
